# Identification of Survival-Associated Alternative Splicing Signatures in Lung Squamous Cell Carcinoma

**DOI:** 10.3389/fonc.2020.587343

**Published:** 2020-09-30

**Authors:** Yang Liu, Wenxiao Jia, Ji Li, Hui Zhu, Jinming Yu

**Affiliations:** ^1^Lung Cancer Center, West China Hospital, Sichuan University, Chengdu, China; ^2^Department of Radiation Oncology, Shandong Cancer Hospital and Institute, Shandong First Medical University and Shandong Academy of Medical Sciences, Jinan, China; ^3^Department of Oncology, Renmin Hospital of Wuhan University, Wuhan, China

**Keywords:** alternative splicing, TCGA, lung squamous cell carcinoma, prognosis, splicing factors

## Abstract

**Purpose:** Alternative splicing (AS) is a post-transcriptional process that plays a significant role in enhancing the diversity of transcription and protein. Accumulating evidences have demonstrated that dysregulation of AS is associated with oncogenic processes. However, AS signature specifically in lung squamous cell carcinoma (LUSC) remains unknown. This study aimed to evaluate the prognostic values of AS events in LUSC patients.

**Methods:** The RNA-seq data, AS events data and corresponding clinical information were obtained from The Cancer Genome Atlas (TCGA) database. Univariate Cox regression analysis was performed to identify survival-related AS events and survival-related parent genes were subjected to Gene Ontology enrichment analysis and gene network analysis. The least absolute shrinkage and selection operator (LASSO) method and multivariate Cox regression analysis were used to construct prognostic prediction models, and their predictive values were assessed by Kaplan-Meier analysis and receiver operating characteristic (ROC) curves. Then a nomogram was established to predict the survival of LUSC patients. And the interaction network of splicing factors (SFs) and survival-related AS events was constructed by Spearman correlation analysis and visualized by Cytoscape.

**Results:** Totally, 467 LUSC patients were included in this study and 1,991 survival-related AS events within 1,433 genes were identified. *SMAD4, FOS, POLR2L*, and *RNPS1* were the hub genes in the gene interaction network. Eight prognostic prediction models (seven types of AS and all AS) were constructed and all exhibited high efficiency in distinguishing good or poor survival of LUSC patients. The final integrated prediction model including all types of AS events exhibited the best prognostic power with the maximum AUC values of 0.778, 0.816, 0.814 in 1, 3, 5 years ROC curves, respectively. Meanwhile, the nomogram performed well in predicting the 1-, 3-, and 5-year survival of LUSC patients. In addition, the SF-AS regulatory network uncovered a significant correlation between SFs and survival-related AS events.

**Conclusion:** This is the first comprehensive study to analyze the role of AS events in LUSC specifically, which improves our understanding of the prognostic value of survival-related AS events for LUSC. And these survival-related AS events might serve as novel prognostic biomarkers and drug therapeutic targets for LUSC.

## Introduction

Lung cancer is the leading cause of cancer-related deaths across the world, and non-small cell lung cancer (NSCLC) is the most prevalent subtype, accounting for more than 80% of all cases (1–3). NSCLC can be divided into lung squamous cell carcinoma (LUSC) and non-squamous cell carcinoma, including adenocarcinoma (LUAD), large cell carcinoma (LCC) and NSCLC-not otherwise specified (4). In recent years, although significant progress has been achieved in treating NSCLC, such as immunotherapy and targeted therapy, the prognosis of advanced LUSC is still poor, and the 5-year survival rate is <5% (5, 6). This can be interpreted by the lower incidence of epidermal growth factor receptor (EGFR) mutations and ALK rearrangements in LUSC patients when compared with LUAD patients. In addition, patients with LUSC are usually diagnosed at an advanced stage, and the preferred treatments are still traditional chemoradiotherapy (7). Thus, given little improvement has been achieved in the treatment and survival for LUSC patients, developing novel biomarkers is needed to improve early diagnosis rate and predict prognosis effectively.

Alternative splicing (AS) is a post-transcriptional process in which a single pre-mRNA is spliced into different arrangements and specific exons are selectively included or excluded to produce various messenger RNA (mRNA) isoforms (8–10). In fact, more than 90% of human genes experience AS, a mechanism that enhances protein diversity and complexity, resulting in the production of nearly 2 million protein molecules with only around 20,000 protein-coding genes (11–13). Besides, AS also regulate the reduced generation of mRNA isoforms by introducing a premature termination codon that leads to mRNA degradation (14). Therefore, different AS events are closely related to protein functions. And accumulating evidences have demonstrated that dysregulation of AS is associated with many human diseases, especially cancer. Accordingly, AS variants are involved in carcinogenesis, including proliferation, invasion, metastasis, apoptosis, and immune escape (15–20). The profiling of AS signature may provide potential novel molecular biomarkers for the diagnosis, treatment, and prognosis of cancers (21–23). In addition, AS events are regulated by splicing factors (SFs), and abnormal expression alternations of SFs may lead to global changes of AS events in cancer. Specifically, in some cases of malignant tumors, the dysregulation of SFs, acting as oncogenes, can give rise to a specific AS isoforms of cancer promotion (24–26). Therefore, exploring the potential regulatory network between AS and SFs may provide a new insight of mechanisms of oncogenesis and development of malignant tumors.

In recent years, cancer-specific AS events have been identified by comparing cancer tissues with normal controls in several studies. And associations between AS signatures and overall survival (OS) time of patients have been systematically evaluated in a variety of cancers, such as colorectal cancer, ovarian cancer, breast cancer, kidney renal clear cell carcinoma, esophageal carcinoma as well as NSCLC (26–32). However, although systematic analysis and prognostic prediction models of AS events for NSCLC have been conducted in some studies, no comprehensive study specifically for LUSC has been performed.

In this study, we conducted a systematic analysis of AS events in patients with LUSC by using The Cancer Genome Atlas (TCGA) database and identified a number of survival-associated AS events. Furthermore, a survival-predicting model was constructed to evaluate the prognostic value of AS signatures in LUSC. And we also uncovered underlying function pathways and gene interaction networks of corresponding genes. In addition, a SF-AS regulatory network was constructed to provide a new perspective to understand the correlation between SFs and AS events.

## Materials and Methods

### Data Collection and Processing

The RNA-seq data and corresponding clinical information of the LUSC cohort were downloaded from TCGA data portal (https://portal.gdc.cancer.gov/). AS events data were obtained from the TCGA SpliceSeq database (https://bioinformatics.mdanderson.org/TCGASpliceSeq/), including seven types: alternate acceptor site (AA), alternate donor site (AD), alternate promoter (AP), alternate terminator (AT), exon skip (ES), mutually exclusive exons (ME), and retained intron (RI), and the intersections among these AS types were presented by the Upset plot (33). The percent spliced index (PSI) value, which is an intuitive ratio ranging from 0 to 1, was used to quantify seven types of AS events. To generate a reliable set, only AS events that met the screening criteria (percentage of samples with PSI value ≥ 75%, average PSI value ≥ 0.05 and standard deviation of PSI value ≥ 0.01) were included in this study. A unique identifier that combines gene symbol, ID number in the SpliceSeq database and splicing pattern was used to present each AS event. Patients with a survival time <30 days or no corresponding data of RNA-seq or AS events were excluded, and a total of 467 LUSC patients were eventually selected in our study cohort.

### Identification of Survival-Related AS Events, Gene Ontology (GO) Analysis and Gene Network Construction

To evaluate the association between AS events and OS, we performed univariate Cox regression analysis to identify survival-related AS events by using “survival” and “survminer” R packages. And then the Upset plot and volcano plot were exploited to depict these AS events. Furthermore, the bubble plots were used to illustrate the top 20 survival-related AS events for seven types (if more than 20). Then, in order to explore the potential mechanisms of AS events in LUSC, the corresponding genes of survival-related AS events were subjected to Gene Ontology (GO) enrichment analysis, including biological process (BP), cellular component (CC), and molecular function (MF), which was conducted using ClusterProfiler and org.Hs.eg.db packages of R software v.3.6.3 (34). Adjusted *P* < 0.05 was considered statistically significant. In addition, we chose the corresponding genes of most significant survival-related AS events whose univariate regression *P* < 0.01 to construct gene interaction network by the Cytoscape's Reactome FI plugin and determined top 10 hub genes according to the number of connections (35).

### Construction of Prognostic Prediction Models

The least absolute shrinkage and selection operator (LASSO) regression analysis was performed to select most significant AS events out of all the OS-related AS events, which were identified in the above univariate Cox analysis (*p* < 0.05) (36). This process was performed using glmnet R package to avoid model overfitting, and several optimal AS events with non-zero coefficients were identified. Then these most significant AS events in seven types were used to construct prognostic prediction models by multivariate Cox regression analysis, respectively. Eventually, the final prediction model was constructed by integrating all AS events from above seven prediction models.

### Evaluation of the Prediction Models and Construction of Prognostic Nomogram

All LUSC patients were divided into high-risk and low-risk groups according to the median risk score, and Kaplan–Meier (K-M) analysis was conducted to estimate the survival probabilities between two groups. Log-rank tests were used to compare the difference in survival. In order to validate the predictive accuracy of each model, we performed receiver operating characteristic (ROC) curve analysis of 1, 3, 5 years and calculated the value of the area under the curve (AUC). The “survival,” “survminer,” and “survival ROC” packages in R were used to perform survival analyses. Furthermore, samples are reordered according to the risk score, and then the risk score curves, the distribution of survival status, and expression heatmap were generated. And we also evaluated the prognostic role of risk score and other clinicopathological characteristics, such as age, gender, and TNM stage. Then a nomogram based on the risk score of the final prognostic model and clinicopathological variables was established to estimate the individualized survival risk of LUSC patients by using the ‘rms’ package. And the calibration curves for 1-, 3-, and 5-year survival were plotted to assess the predictive accuracy of the nomogram.

### Construction of the Potential SF-AS Regulatory Network

We further obtained the SFs data from the SpliceAid 2 database and then conducted Spearman correlation analysis to investigate the correlation between PSI values of survival-related AS events and the expression levels of SF genes. *P* < 0.001 and the absolute value of correlation coefficient > 0.5 were considered statistically significant. The potential SF-AS regulatory network was constructed and visualized by Cytoscape software (version 3.7.2).

### Statistical Analysis

The risk score of each LUSC patients was calculated according to the PSI value of each AS events and the corresponding regression coefficient, which was obtained from multivariate Cox regression analysis. The formula was as follows: $Riskscore=\overset{n}{\underset{i}{\sum }}PSIi\times \beta i$, where β represents the regression coefficient. And the prognostic role of risk score and other clinicopathological characteristics was evaluated by univariate and multivariate Cox regression analysis. All statistical analysis was performed in the R software (version 3.6.3). Two-sided *P* < 0.05 was considered statistically significant.

## Results

### Clinical Characteristics and Integrated AS Events Profiles in LUSC Cohort

**Figure 1:**
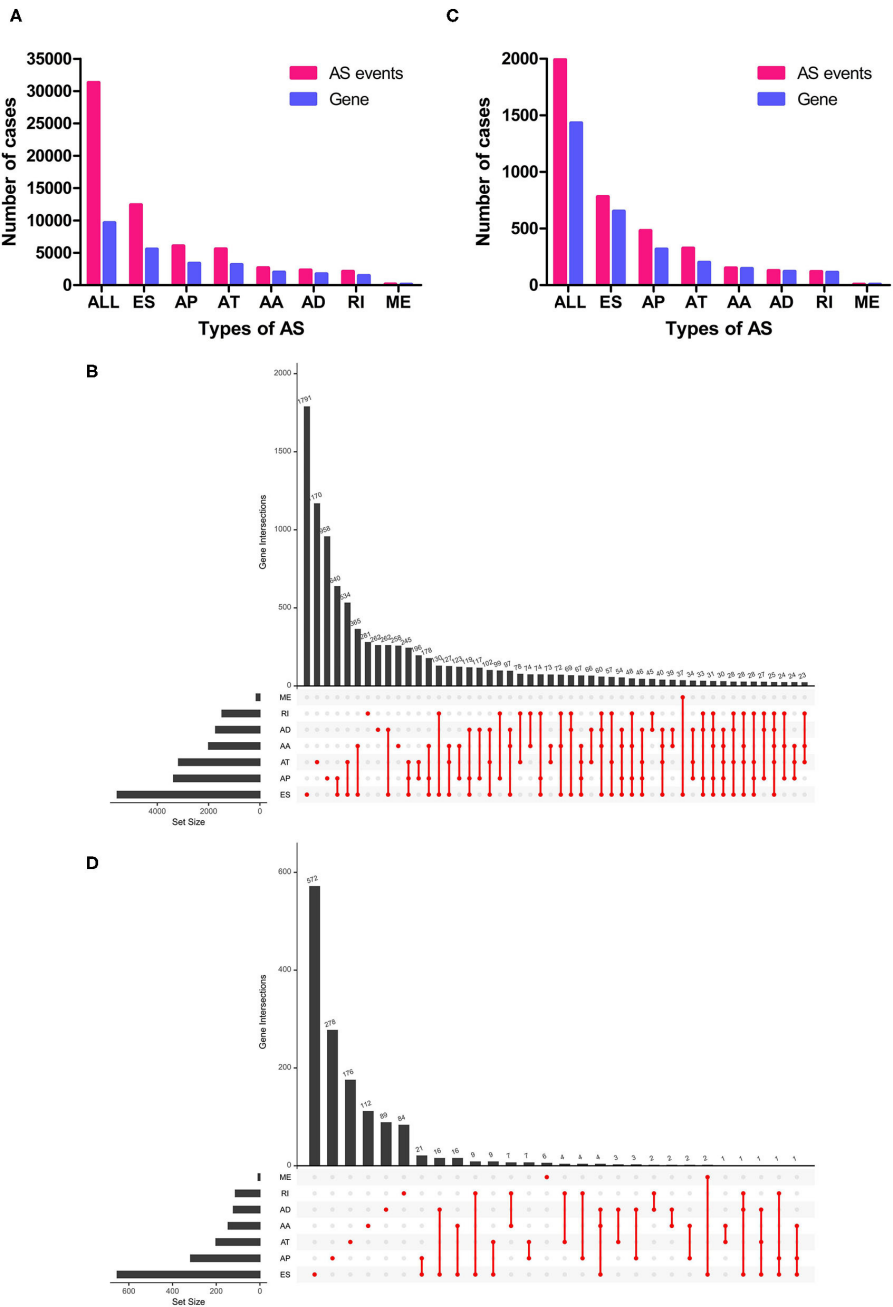
Overview of AS events in LUSC patients. **(A)** Numbers of AS events and parent genes for each AS type in LUSC patients. **(B)** Upset plot of interactions among seven types of all AS events in LUSC patients. **(C)** Numbers of survival-related AS events and parent genes for each AS type in LUSC patients. **(D)** Upset plot of interactions among seven types of survival-related AS events in LUSC patients. AS, alternative splicing; LUSC, lung squamous cell carcinoma.

In total, 467 LUSC patients (346 men and 121 women) from TCGA database were included in the present study, and these patients' characteristics and clinical information were listed in Table 1. Most patients were in early stages with 222 (47.54%) I, 154 (32.98%) II, 81 (17.34%) III, and 6 (1.28%) stage IV. In this cohort, a total of 31,323 AS events in 9,645 parent genes were detected, including 12,400 ESs in 5,556 genes, 6,065 APs in 3,359 genes, 5,578 ATs in 3,166 genes, 2,680 AAs in 1,997 genes, 2,324 ADs in 1,724 genes, 2,127 RIs in 1,474 genes, and 149 MEs in 143 genes (Figure 1A). The intersections among these seven types of AS events were showed in the Upset plot, and these results indicated that one single gene might have several types of AS events (Figure 1B). In addition, ES was the predominant type in LUSC cohort, followed by AP and AT, whereas ME was the least splicing event.

**Table 1:** Characteristics of patients with LUSC from TCGA database.

**Characteristics**	**No. of patients**	**%**
Age at diagnosis (years)		
<70	259	55.46
≥70	208	44.54
Sex		
Male	346	74.09
Female	121	25.91
Stage		
I	222	47.54
II	154	32.98
III	81	17.34
IV	6	1.28
Unknown	4	0.86
T category		
T1	104	22.27
T2	274	58.67
T3	68	14.56
T4	21	4.50
N category		
N0	295	63.17
N1	124	26.55
N2	39	8.35
N3	5	1.07
Unknown	4	0.86
M category		
M0	387	82.87
M1	6	1.28
Unknown	74	15.85

*LUSC, lung squamous cell carcinoma; TCGA, The Cancer Genome Atlas*.

### Survival-Related AS Events in LUSC

**Figure 2:**
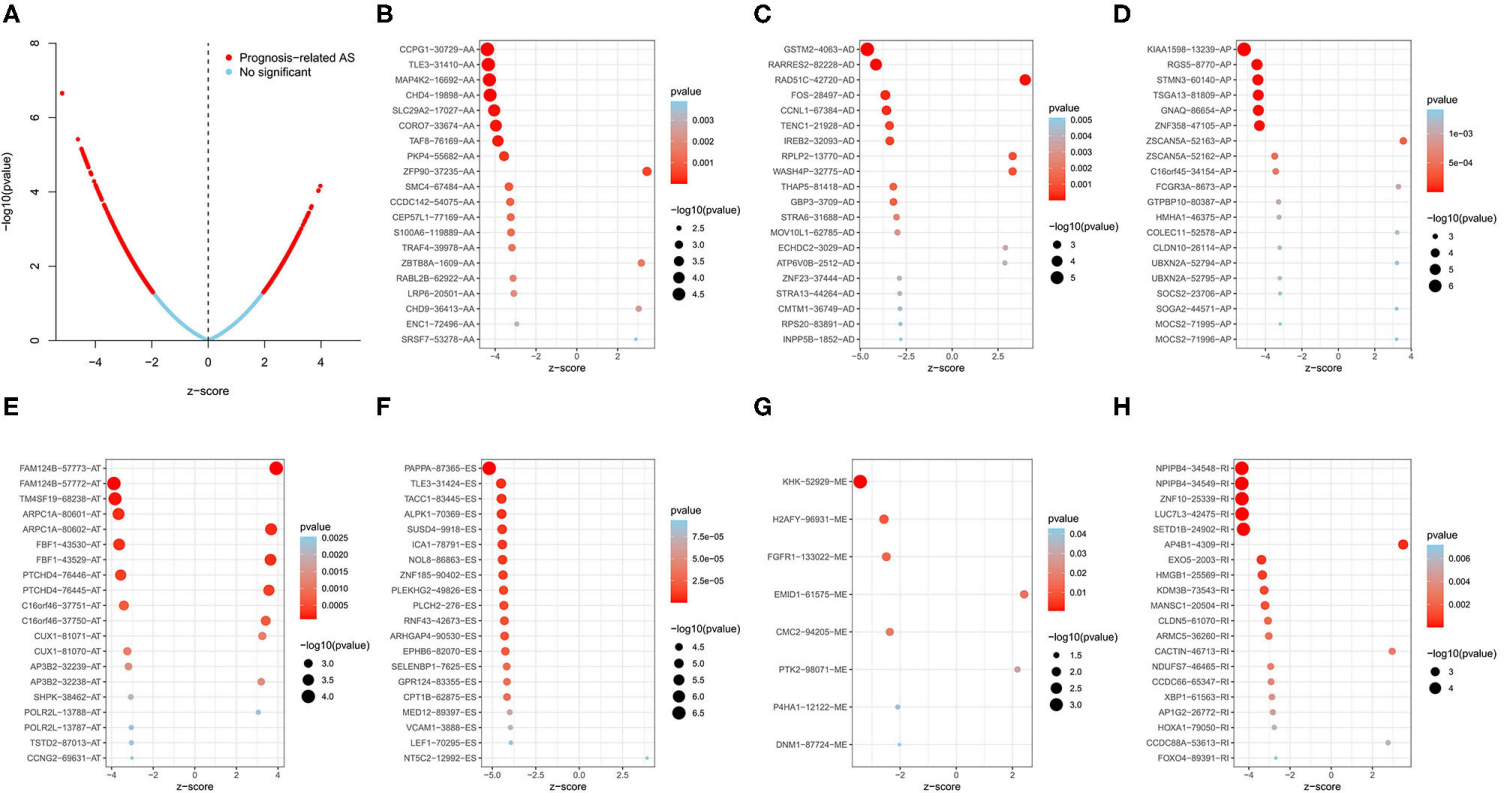
Survival-related AS events in LUSC patients. **(A)** Volcano plot of survival-related AS events (red dot) and survival-irrelated AS events (blue dot). **(B–H)** Bubble plots of top 20 most significant survival-related AS events in AA, AD, AP, AT, ES, ME, and RI, respectively. AA, alternate acceptor site; AD, alternate donor site; AP, alternate promoter; AT, alternate terminator; ES, exon skip; ME, mutually exclusive exons; RI, retained intron.

To investigate the relationship between AS events and OS in LUSC patients, univariate Cox regression analysis was conducted to determine survival-related AS events. As results, a total of 1,991 survival-related AS events in 1,433 parent genes were identified in LUSC cohort (*P* < 0.05), consisting of 780 ESs in 653 genes, 481 APs in 317 genes, 325 ATs in 201 genes, 151 AAs in 145 genes, 127 ADs in 121 genes, 119 RIs in 112 genes, and MEs in 8 genes (Figure 1C and Supplementary Table 1). And the Upset plot was generated to visualize the intersecting sets, and it revealed that one gene could have up to three survival-related AS events (Figure 1D). For instance, ES, AA, and AD events in *ATXN2L* (ataxin 2 like) gene were all significantly associated with OS in LUSC patients. The distribution of these AS events was also displayed in the volcano plot (Figure 2A). In addition, the top 20 most significant survival-related AS events (if available) for each splice type were visualized in bubble plots, and the results showed that most of these events were favorable prognostic factors [hazard ratio (HR) < 1, *P* < 0.05] (Figures 2B–H).

### GO Analysis and Interaction Network for Parent Genes of Survival-Related AS Events

**Figure 3:**
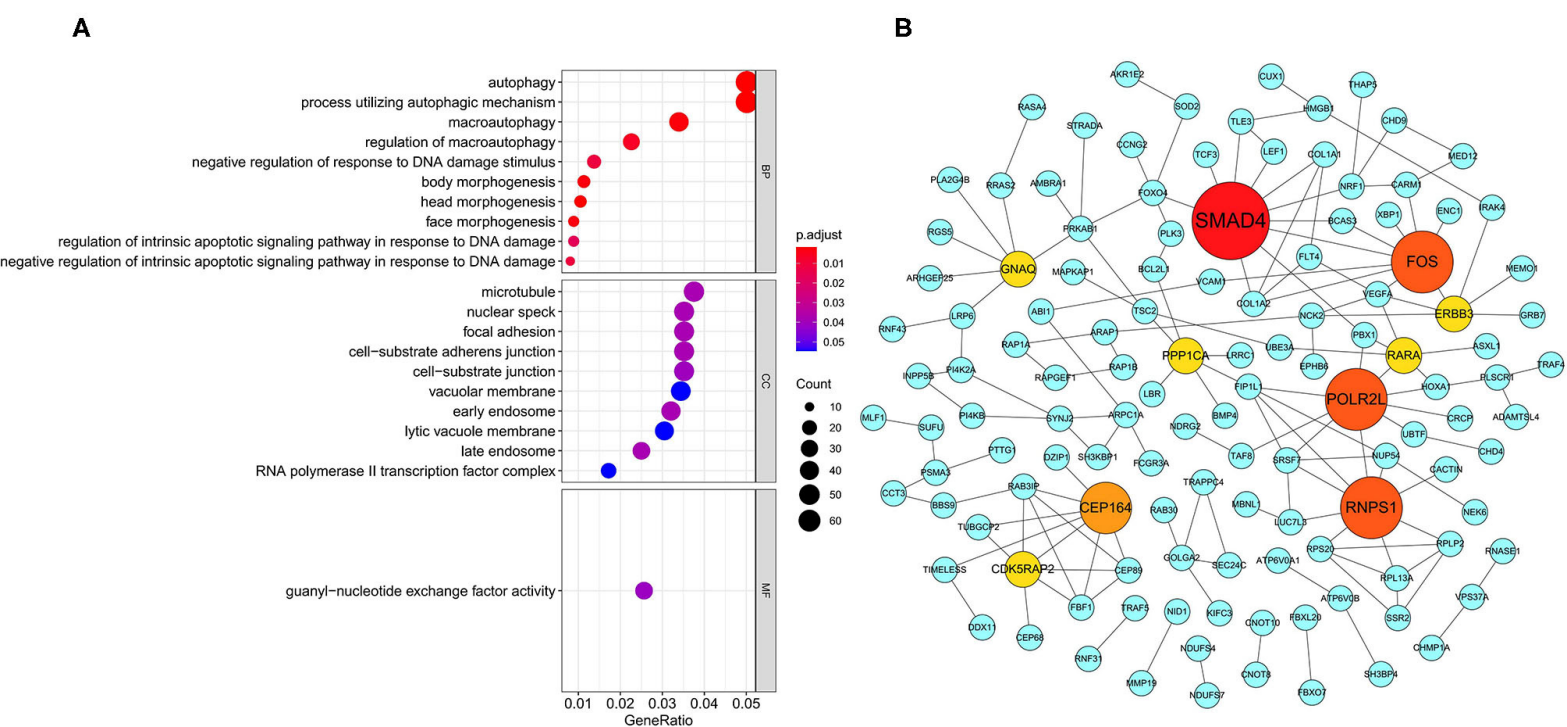
GO enrichment analysis and gene interaction network. **(A)** GO enrichment analysis (BP/CC/MF) of the parent genes from survival-related AS events. **(B)** Gene interaction network of the most significantly survival-related genes. GO, Gene Ontology; BP, biological process; CC, cellular component; MF, molecular function.

In order to explore the potential mechanisms of survival-related AS genes, we performed GO enrichment analysis by using 1,433 parent genes from 1,991 OS-related AS events. The BP terms of these genes were significantly enriched in “autophagy,” “process utilizing autophagic mechanism,” and “macroautophagy.” “Microtubule,” “nuclear speck,” and “focal adhesion” were the three most enriched CC terms, and “guanyl–nucleotide exchange factor activity” was the only significant MF term (Figure 3A). The most significantly survival-associated genes in LUSC (*P* < 0.01) were uploaded to Cytoscape to generate a gene interaction network, which was shown in Figure 3B. Several hub genes were identified, such as *SMAD4* (SMAD family member 4), *FOS* (Fos proto-oncogene), *POLR2L* (RNA polymerase II, I and III subunit L), and *RNPS1* (RNA binding protein with serine rich domain 1).

### Construction and Evaluation of the Prognostic AS Models and Nomogram for LUSC Patients

**Figure 4:**
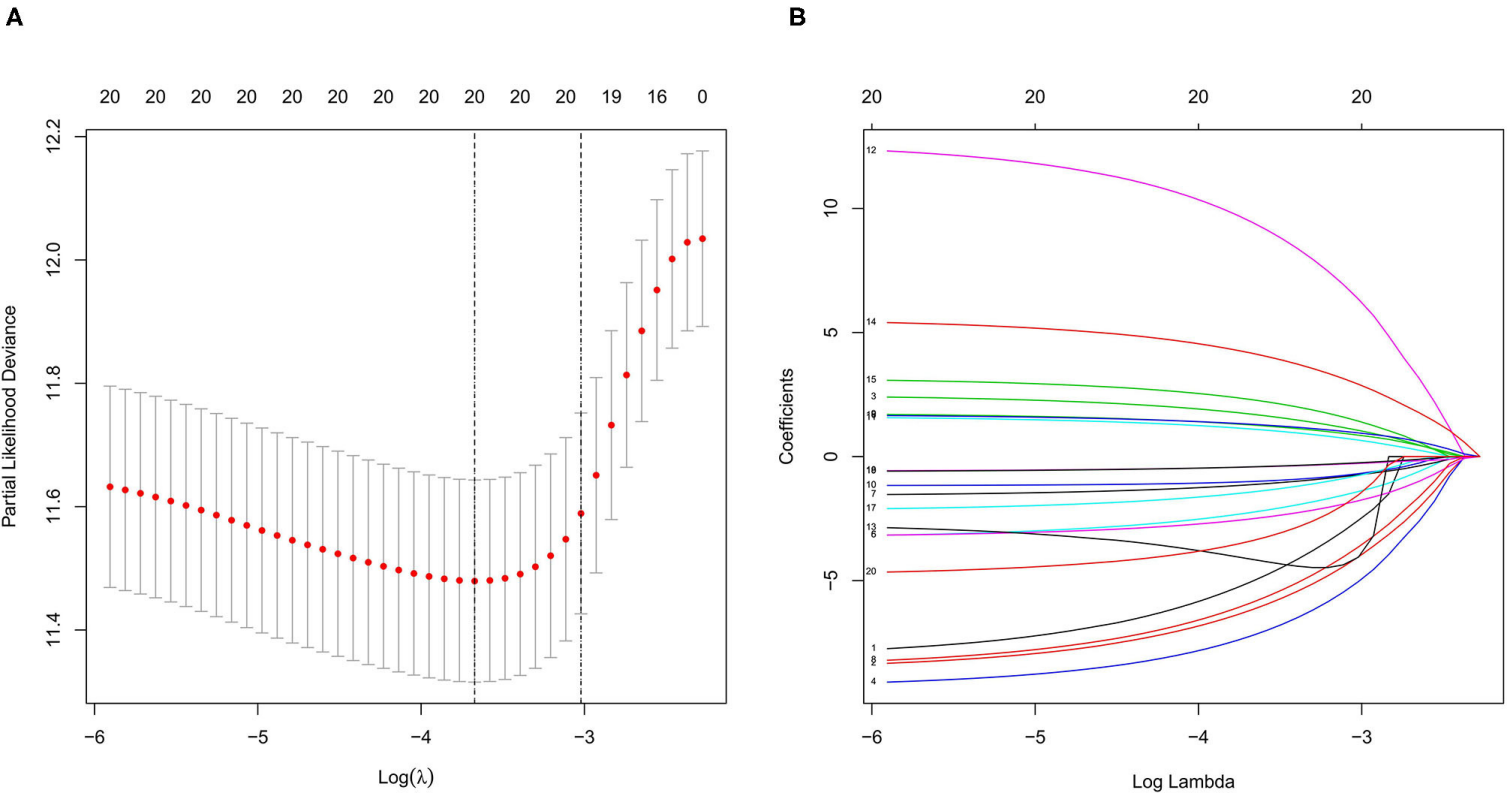
LASSO regression analysis of survival-related AS events (all). **(A)** Cross-validation for the selection of optimal parameter (lambda) and dotted vertical lines were drawn at the optimal values. **(B)** LASSO coefficient profiles of the candidate survival-related AS events. AS events with non-zero coefficients were identified by the optimal lambda. LASSO, least absolute shrinkage and selection operator.

**Figure 5:**
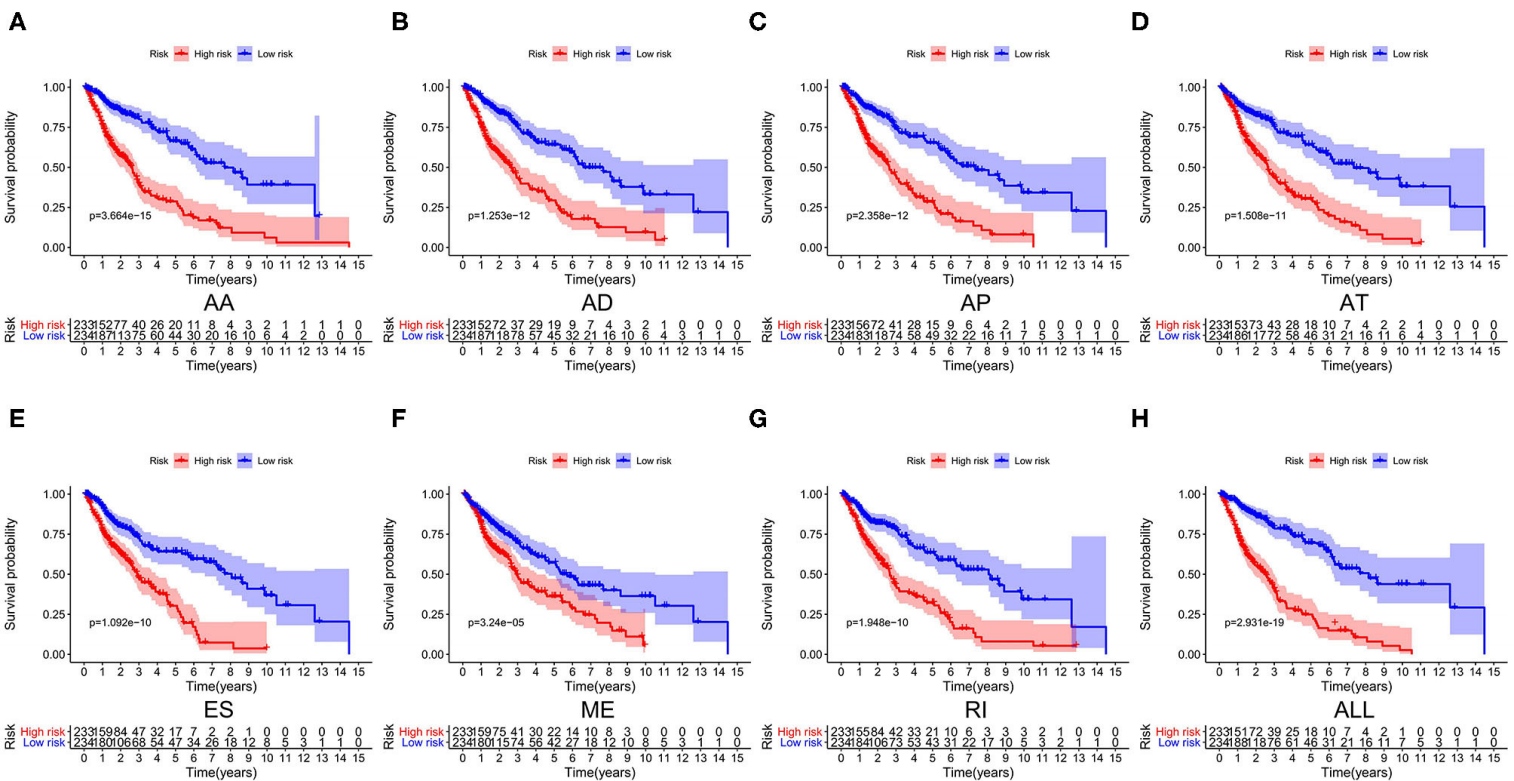
Kaplan–Meier survival analyses of eight prognostic models in LUSC. **(A–H)** Kaplan–Meier curves of prognostic models constructed with AS events of AA, AD, AP, AT, ES, RI, ME and all splicing types. AA, alternate acceptor site; AD, alternate donor site; AP, alternate promoter; AT, alternate terminator; ES, exon skip; ME, mutually exclusive exons; RI, retained intron.

**Figure 6:**
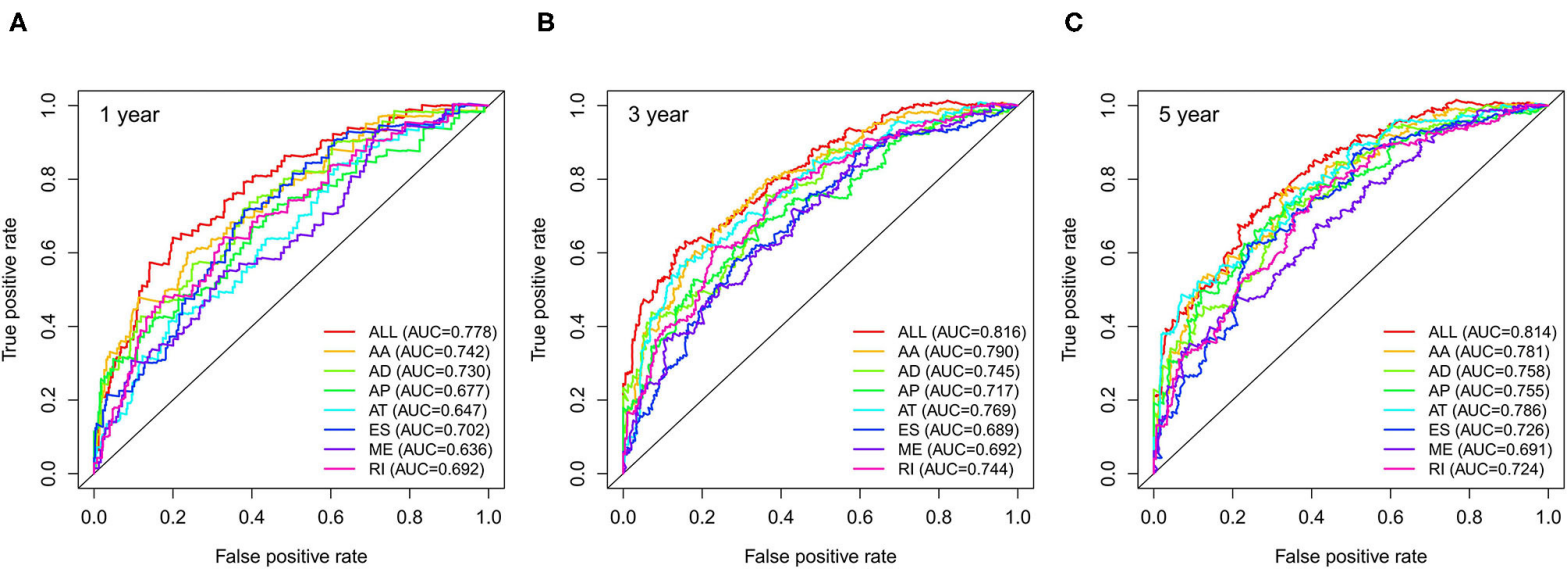
ROC curves with AUCs of eight prognostic models in 1 year **(A)**, 3 years **(B)** and 5 years **(C)**. ROC, receiver operating characteristic.

**Figure 7:**
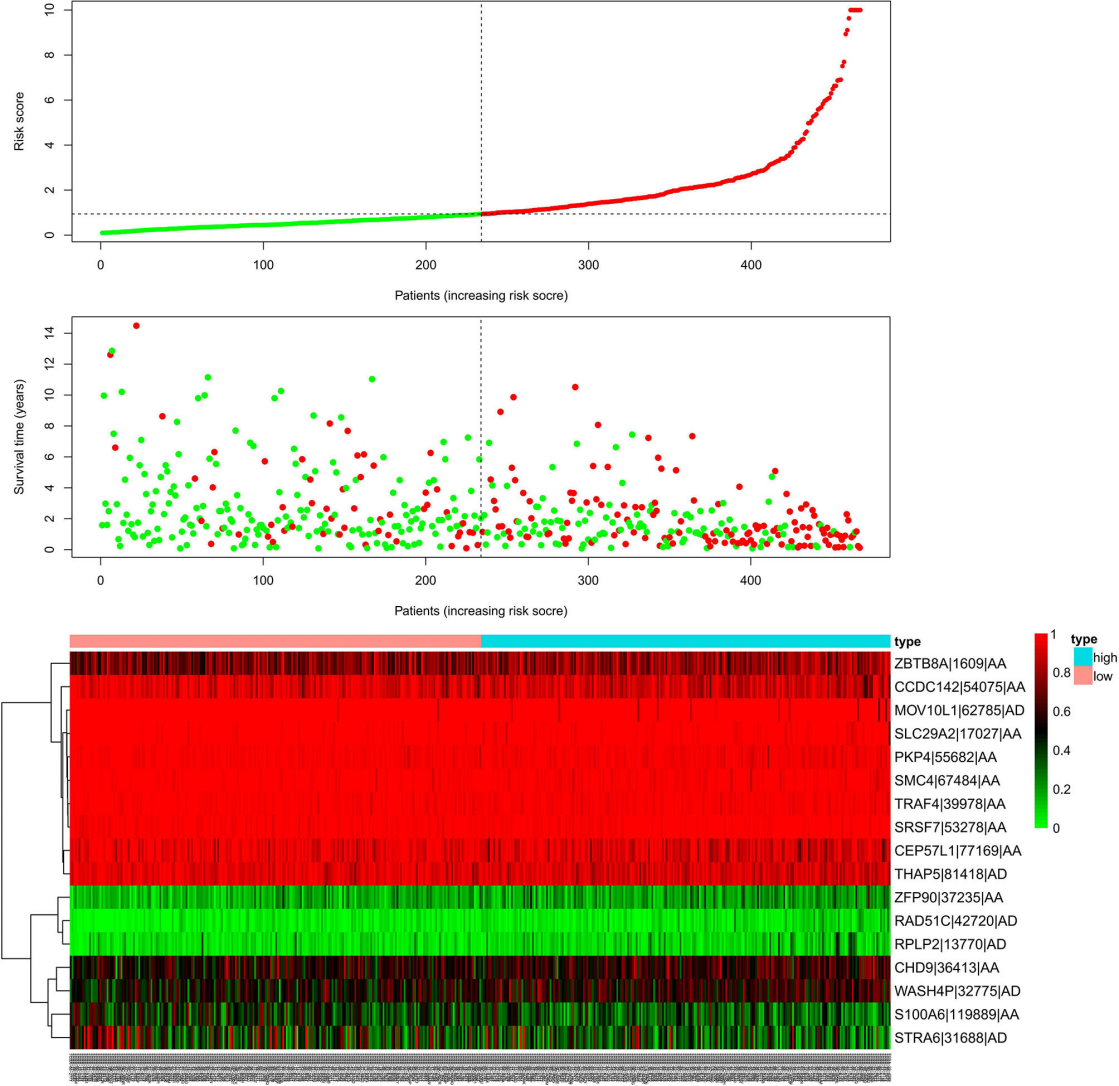
Risk scores analysis of the final prognostic model. The upper part represents the risk score curves, the middle part indicates the distribution of patients' survival time and status, and the bottom shows the PSI value heatmap for the final prognostic model. PSI, percent spliced index.

**Figure 8:**
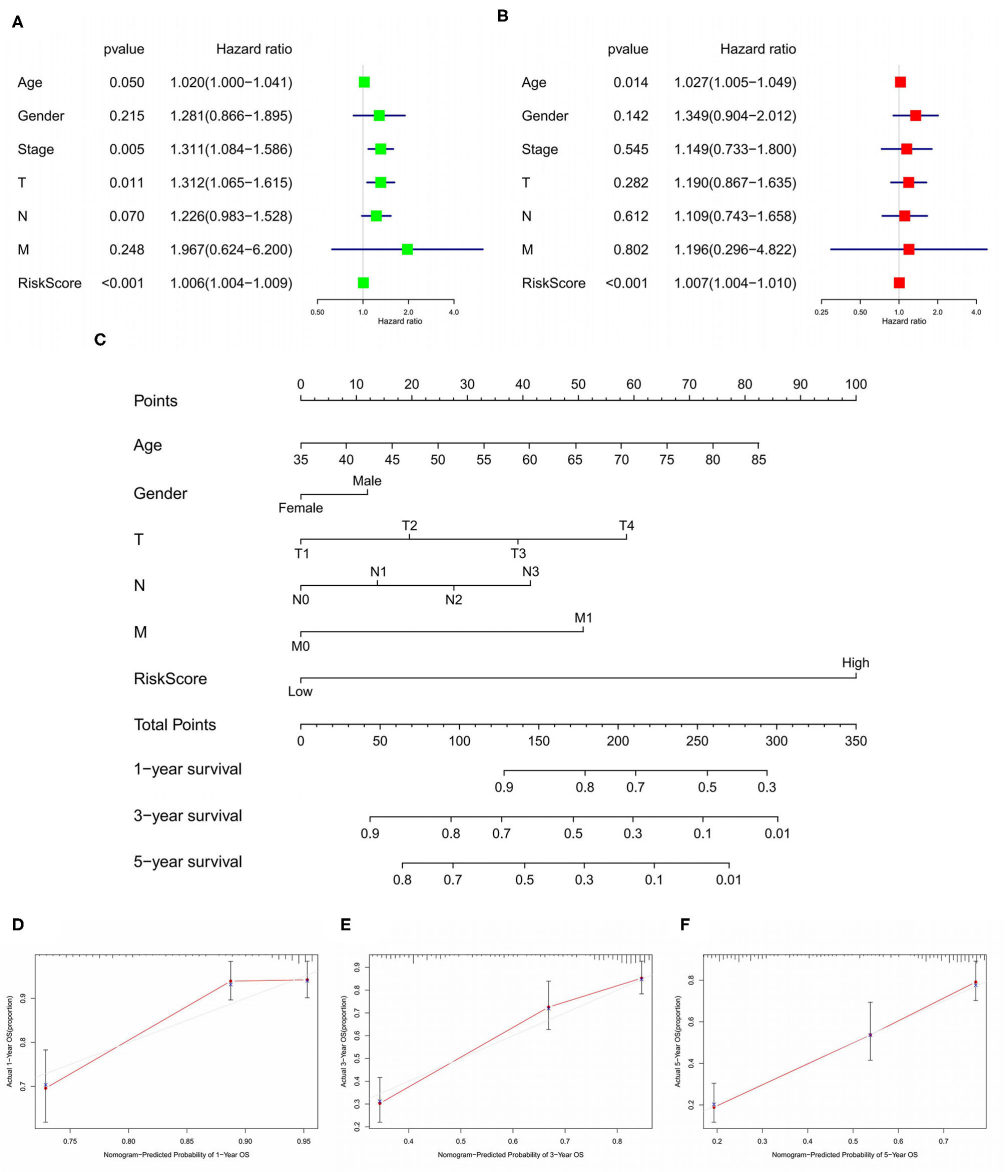
Forest plots of Cox regression analysis for evaluating the independent prognostic value of the risk score (All) based on univariate analysis **(A)** and multivariate analysis **(B)**. Nomogram for predicting 1-, 3-, and 5-year OS of LUSC patients **(C)**. Calibration curves for the survival probability of 1, 3, or 5 years **(D–F)**. OS, overall survival; LUSC, lung squamous cell carcinoma.

We used LASSO regression analysis to select the most significant AS events in seven types, which were used as candidates to build prognostic prediction models by multivariate Cox regression analysis (Supplementary Table 2 and Supplementary Figure 1). And all these AS events were further analyzed to construct the final prediction model (all types) (Table 2 and Figure 4). Then LUSC patients were divided into high-risk and low-risk groups based on the median value of the risk score. As shown in Figure 5, K-M survival analysis revealed that all of the eight prediction models exhibited significant power in distinguishing good or poor survival of LUSC patients with all *P* < 0.05. Moreover, in order to evaluate the prediction efficiency of these prognostic models, the ROC curve of 1, 3, and 5 years were applied, which revealed that all the models exhibited a strong performance to predict the survival of LUSC patients with AUC values ranging from 0.636 to 0.816. And compared with other models built on individual types of AS events, the final integrated prediction model performed the greatest prognostic power with the maximum AUC values of 0.778, 0.816, 0.814 in 1, 3, 5 years ROC curves, respectively (Figure 6). Meanwhile, the risk score curves, the distribution of survival status, and the PSI value heatmap for these prognostic models were displayed (Figure 7 and Supplementary Figure 2). In addition, we evaluated the prognostic value of risk score and other clinicopathological characteristics, such as age, gender, and TNM stage, by conducting univariate and multivariate Cox regression analysis, and the risk score (All) served as an independent prognostic factor for LUSC patients in multivariate analysis after other clinicopathological characteristics were adjusted (HR = 1.007, 95% CI: 1.004 1.010, *P* < 0.001) (Figures 8A,B). Finally, we built a nomogram to predict the 1-, 3-, and 5-year OS of LUSC patients based on the risk score (All) and clinicopathological parameters including age, gender, and TNM stage (Figure 8C). The calibration curves for the survival probability of 1, 3, or 5 years showed good uniformity between the prediction and the actual observation (Figures 8D–F).

**Table 2:** Information of AS events used for construction of the final prognostic model.

**Gene symbol**	**Spliceseq ID**	**AS type**	**coef**	**HR**	**HR.95L**	**HR.95H**	***P*-value**
SLC29A2	17027	AA	−9.269156134	9.43E-05	5.61E-07	0.015834179	0.000391408
PKP4	55682	AA	−9.290847632	9.23E-05	6.58E-08	0.129468824	0.011974724
ZFP90	37235	AA	2.48074144	11.95012143	2.02900869	70.38185836	0.006105946
SMC4	67484	AA	−9.893859995	5.05E-05	6.14E-08	0.04152125	0.003865157
CCDC142	54075	AA	−3.603432799	0.027230086	0.003683186	0.201314186	0.000415028
CEP57L1	77169	AA	−3.333966231	0.035651423	0.00509991	0.249224754	0.000778393
S100A6	119889	AA	−1.689089325	0.184687638	0.063747265	0.535074302	0.001857083
TRAF4	39978	AA	−8.757648918	0.000157254	8.87E-08	0.278819849	0.02175595
ZBTB8A	1609	AA	1.998526165	7.378173873	1.661840432	32.75732656	0.008593236
CHD9	36413	AA	1.708103349	5.518484905	1.538196465	19.79830038	0.008777044
SRSF7	53278	AA	11.81296344	134991.0063	6.101583046	2986531799	0.02065256
RAD51C	42720	AD	5.687489273	295.1516444	16.73215293	5206.412681	0.000102813
RPLP2	13770	AD	3.121537314	22.68122101	3.523660347	145.9952821	0.001017282
WASH4P	32775	AD	1.752303826	5.767875561	1.770594167	18.78939234	0.00363613
THAP5	81418	AD	−2.413177611	0.089530349	0.005239216	1.529939451	0.095646266
STRA6	31688	AD	−0.743422958	0.475483566	0.2250065	1.004791516	0.051481732
MOV10L1	62785	AD	−4.968112301	0.006956267	0.000148067	0.326810036	0.011427475

*AS, alternative splicing; AA, alternate acceptor site; AD, alternate donor site*.

### Potential Regulatory Network of SFs and Survival-Related AS Events

**Figure 9:**
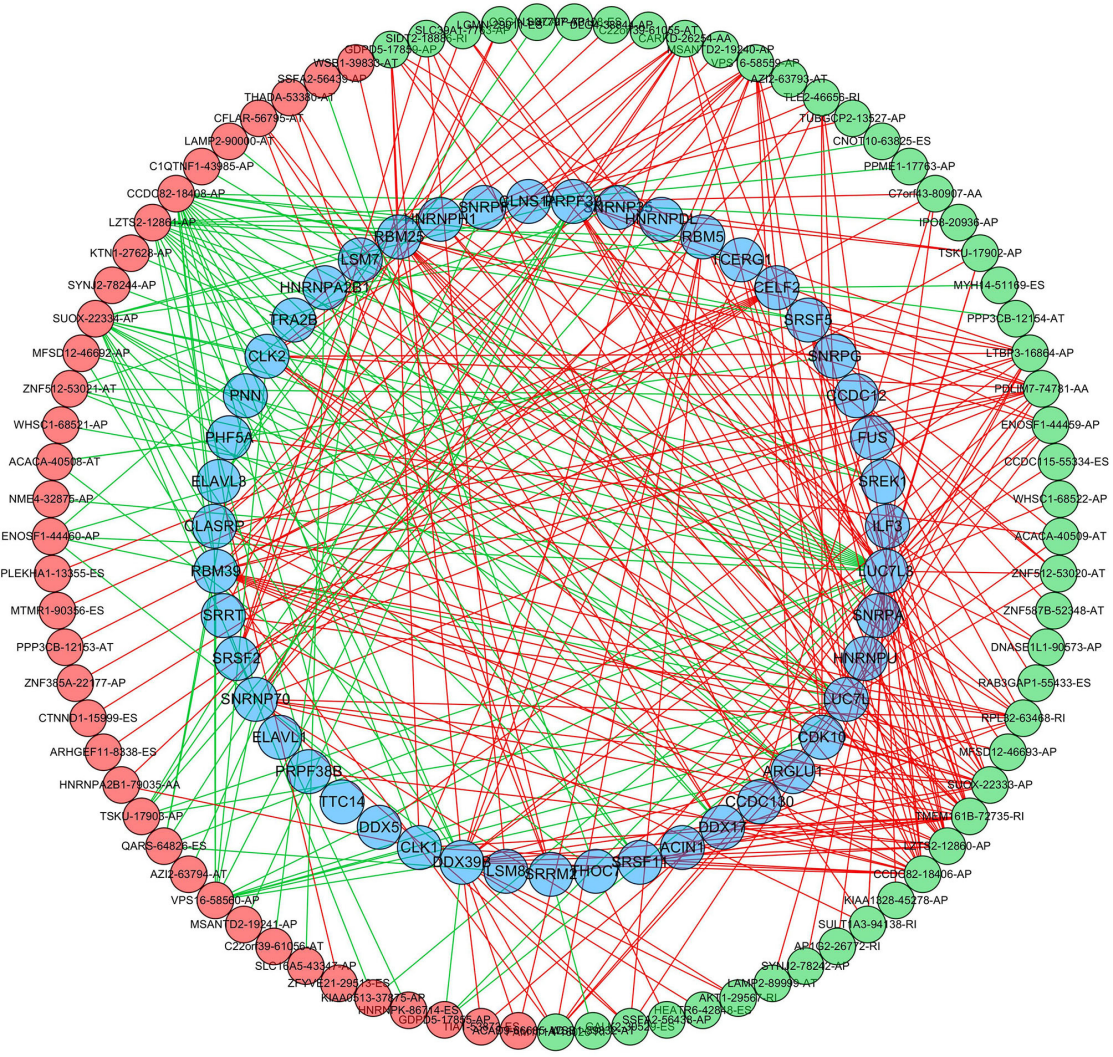
Regulatory network of SFs and survival-related AS events in LUSC. The favorable prognosis AS events (green dots) or adverse prognosis AS events (red dots) were positively (red line) or negatively (green line) regulated by the expression of SFs (blue dots). SFs, splicing factors; AS, alternative splicing.

To further explore the potential splicing-regulatory network of survival-related AS events in the LUSC cohort, we collected 390 SFs data from the SpliceAid 2 database. Then the correlations between the PSI values of OS-associated AS events and the gene expression level of SFs were analyzed by Spearman test, and the significantly related pairs with correlation coefficient > 0.5 and *P* < 0.001 were selected to construct the correlation network. As shown in Figure 9, the expression levels of 47 SFs (blue dots) were significantly correlated with 85 survival-related AS events, including 48 favorable prognosis AS events (green dots) and 37 adverse prognosis AS events (red dots). Interestingly, we found that the majority of poor survival prognostic AS events were negatively regulated by SFs (green lines), whereas most AS events with good prognosis were positively correlated with the expression of SFs (red lines).

## Discussion

LUSC accounts for ~30% of all cases of NSCLC and causes about 400,000 deaths per year worldwide (2). Patients with LUSC are often diagnosed with advanced stage and the main treatment modalities are surgery, radiotherapy and chemotherapy. Although lots of progress of molecular targeted agents have been made in the treatment of LUAD, there is a lack of effective targeted treatment options for patients with LUSC since EGFR mutations and ALK fusions are not typically present in LUSC. And the preferred treatments for LUSC patients are still traditional chemoradiotherapy (5, 7). Therefore, to prescribe optimal individualized management and improve the survival rate of LUSC patients, it is of vital importance to identify effective prognostic biomarkers with high sensitivity and specificity.

In recent years, with the rapid development of high-throughput sequencing technology, numerous studies have explored the genome-wide prognostic biomarkers of different cancers including LUSC. For instance, Li et al. evaluated the prognostic value of epigenetic process in LUSC and found that the mean level of DNA methylation was significantly lower in LUSC. Meanwhile, they also identified that four methylation-driven genes, *GCSAM, GPR75, NHLRC1*, and *TRIM58*, could be served as prognostic indicators for LUSC (37). In addition, many other studies have focused on transcription level analyses, such as studies on mRNAs, long non-coding RNAs (lncRNAs) or microRNAs (38–41).

AS, as a post-transcriptional regulatory process that modifies more than 90% of human genes, plays a significant role in enhancing the diversity of transcription and protein (9, 12, 13). In recent years, the dysregulation of AS has been found to be associated with the occurrence and development of a variety of cancers including NSCLC (26–32). For instance, Li et al. profiled the genome-wide AS events of NSCLC from TCGA and constructed prognostic predictors for LUAD, LUSC and merged NSCLC patients. Although these prediction models showed a high predictive performance, the potential mechanism of the relationship between AS events and NSCLC has not been deeply discussed (26). In addition, Zhao et al. carried out an AS analysis in NSCLC from the perspective of different sexes and subtypes, and prognostic models and SF-AS network were constructed to reveal the mechanism of AS events affecting the prognosis of NSCLC (32). However, there are few systematic works have been devoted to investigate the role of AS events in LUSC specifically.

In this study, we performed a systematic analysis of the AS signatures in 467 LUSC patients from TCGA. A total of 31,323 AS events in 9,645 parent genes were identified, of which 1,991 AS events in 1,433 genes were significantly associated with the survival of LUSC patients. To further uncover the potential functions of these AS events, GO enrichment analysis was performed and autophagy was the most enriched process among BP pathways. Autophagy is a biological process that captures intracellular proteins and organelles and degrades them in lysosomes, and the degradation products can be released into the cytoplasm for recycling, which plays a significant role in preventing the toxic accumulation of damaged proteins and organelles, maintaining the homeostasis of metabolism and energy, and promoting the survival of cells in starvation (42–44). Nowadays, autophagy has been widely explored, and its functions and mechanisms in oncogenesis, tumor progression and resistance to anticancer therapy have been gradually revealed (45–48). Moreover, autophagy is essential in NSCLC, which is consistence with our results (49, 50).

And as one of the key structures of cytoskeleton, microtubule plays an important role in many cellular functions, such as mitosis, cellular signal transduction, intracellular substance transport, and maintaining normal cell morphology (51, 52). Microtubules and tubulins are important targets for cancer therapy and several microtubule-targeted chemotherapeutic drugs, such as vinorelbine, paclitaxel, and docetaxel, can break the balance between microtubules and tubulins, and then affect the normal cell cycle progression and lead to the death of tumor cells eventually (52–55). From the GO analysis, microtubule was significantly enriched in CC terms, indicating that abnormal AS events might be associated with the drug sensitivity and resistance of LUSC, which needs to be approved by further studies. In addition, our GO analysis shows that several top significant CC terms were closely related to cell adhesion. In fact, the relationship between cell adhesion and tumor has long been studied, and aberrant cell relationship can promote tumor progression and metastasis (56, 57). AS events might influence the occurrence and development of LUSC by regulating cell adhesion functions.

Furthermore, guanyl–nucleotide exchange factor activity was the only significant MF in the LUSC cohort. Rho GTPases, members of Ras superfamily, can be positively activated by guanyl-nucleotide exchange factors and then interact with downstream signaling to regulate a variety of cellular functions, such as cell motility, cell adhesion and cell proliferation (58–60). Recent studies indicate that Rho GTPase signaling is dysregulated in many cancers, which is closely related to cancer development and malignant phenotypes, including migration, invasion, metastasis, and drug resistance (61, 62). From what has been mentioned above, survival-related AS events might potentially influence various pathophysiological processes to regulate the occurrence and development of LUSC, which needs to be confirmed by further researches.

We also established a gene interaction network based on the most significantly associated survival genes in LUSC (*P* < 0.01), and several hub genes were identified, such as *SMAD4* and *FOS*. *SMAD4* gene is a tumor suppressor gene, and its mutations have been found in a variety of cancers, such as pancreatic cancer, colorectal cancer and lung cancer. The loss of *SMAD4* expression could affect the progression and treatment of cancers (63–66). And SMAD4, the protein encoded by *SMAD4* gene, acts as a tumor suppressor, mainly by serving as the core mediator of transforming growth factor-beta (TGF-β) signaling pathway. The TGF-β/SMAD4 signaling pathway, which interacts with other classical pathways, such as MAPK, PI3K/AKT, and WNT/β-catenin, can influence cancer initiation and progression through multiple mechanisms, such as apoptosis, DNA damage repair and epithelial-mesenchymal transition (67, 68). The c-FOS protein, encoded by *FOS* gene, is a leucine zipper protein that can dimerize with JUN family proteins to form transcription factor complex AP-1, which in turns to regulate downstream gene expression by binding to specific DNA segments. Previous studies have revealed that c-FOS can mediate multiple aspects of cancers, including proliferation, invasion, metastasis, angiogenesis and apoptosis (69–71). And the expression of c-FOS protein was significantly related with shorter survival times in LUSC patients, indicating our results are reasonable and credible (72).

Furthermore, based on these survival-related AS events, we constructed eight prognostic prediction models (seven types of AS and all AS) using LASSO regression analysis and multivariate Cox regression analysis. Then the K-M analysis and ROC curve analysis were performed to evaluate the predictive value of each model. All of the eight prediction models exhibited high efficiency in distinguishing good or poor survival of LUSC patients, and the final integrated prediction model including all types of AS events exhibited the best prognostic power with the maximum AUC values of 0.778, 0.816, 0.814 in 1, 3, 5 years ROC curves, respectively. In comparison with previous AS models in prognostic prediction for LUSC, the final model in the present study showed higher specificity and reliability. For instance, the prognostic model for LUSC constructed by Li et al. included more than 30 AS events, though those predictors showed a high predictive performance (26). And in the AS analysis performed by Zhao et al., the LUAD and LUSC cohorts were divided into two groups by sex, but the 1 year AUC value of ROC curve in LUSC_MALE group was only 0.752 (32). Furthermore, the risk score in the present model was found to be an independent prognostic factor for LUSC patients, further confirming the accuracy and clinical applicability of the model. And these survival-related AS events in our model could be novel drug therapeutic targets for LUSC treatment in the future.

It is generally acknowledged that AS events are regulated by key SFs, which can identify and bind to cis-regulatory elements of pre-mRNA, thus affecting the selection of exons and splicing sites (73). The mutations of SF gene sequences and the aberrant expression levels of SFs might influence the occurrence of AS events, further leading to the process of oncogenesis (74–76). Thus, we constructed a SF-AS regulatory network to uncover the underlying upstream mechanisms of splicing patterns involved in the survival of LUSC patients. Interestingly, almost all the good survival-related AS events were positively correlated with the expression of SFs, while most adverse prognosis AS variants were negatively regulated by SFs. This result is consistence with those of previous studies, which indicates that SFs might serve as tumor suppressors in LUSC and the downregulation of SFs would give rise to unfavorable prognoses of LUSC patients (26, 28, 30). This splicing correlation network provides a new insight into the regulatory mechanism of AS events in LUSC. However, only surface relationship between SFs and survival-related AS events was uncovered in the present study, and many uncertainties still exist in the detail regulatory mechanisms of SF-AS network in LUSC, which need to be investigated in depth in further studies.

However, there were several limitations in this study. First, this study was conducted only based on the TCGA database with a limited sample size, and no additional external cohort was used to verify the reproducibility of the prediction model. Second, these prognostic models have not been clinically validated, which will be performed by our team using clinical data, especially prospective data, in the future. Finally, since this is a pure bioinformatics analysis, the potential function of survival-related AS events on LUSC and the underlying mechanisms between SFs and AS events have not been clearly elucidated. Further functional biological experiments and clinical studies with larger sample size of LUSC patients are needed to confirm our findings.

To our best knowledge, this is the first comprehensive study to analyze the role of AS events in LUSC specifically. Several survival-related AS events were identified and prognostic prediction models were constructed base on these events. All the models performed well in risk stratification for LUSC patients and the final integrated prediction model exhibited the greatest prognostic power. Moreover, we also constructed a regulatory network between SFs and survival-related AS events, which provides novel insights into the molecular mechanism of splicing patterns in LUSC. Our findings improve our understanding of the prognostic value of survival-related AS events for LUSC, and these SFs and OS-related AS events might be novel drug therapeutic targets for LUSC treatment, which requires to be further studied.

## Data Availability Statement

Publicly available datasets were analyzed in this study. This data can be found here: TCGA data portal (https://portal.gdc.cancer.gov/) and TCGA SpliceSeq database (https://bioinformatics.mdanderson.org/TCGASpliceSeq/).

## Author Contributions

HZ and JY designed the study and provided funding support. YL and WJ performed data analysis of the study. YL and JL contributed to the software analysis. YL prepared the original manuscript. YL, WJ, and JL reviewed and edited the manuscript. All authors contributed to manuscript revision, read, and approved the submitted version.

## Conflict of Interest

The authors declare that the research was conducted in the absence of any commercial or financial relationships that could be construed as a potential conflict of interest.
