# Supplementary material for: Identification of Survival-Associated Alternative Splicing Signatures in Lung Squamous Cell Carcinoma
Source: Front Oncol. 2020 Sep 30;10:587343. doi: 10.3389/fonc.2020.587343 (PMC7561379; doi:10.3389/fonc.2020.587343)
Supplement: Supplementary file 2 [file Table_2.DOCX]

| **Supplementary Table 2. Information of AS events used for construction of prognostic models for seven different types.** | | | | | | | | |
| --- | --- | --- | --- | --- | --- | --- | --- | --- |
| **Type** | **gene symbol** | **Spliceseq ID** | **AS type** | **coef** | **HR** | **HR.95L** | **HR.95H** | **pvalue** |
| AA | SLC29A2 | 17027 | AA | -10.45840128 | 2.87E-05 | 1.93E-07 | 0.004265438 | 4.16E-05 |
|  | PKP4 | 55682 | AA | -6.717838103 | 0.001209149 | 1.17E-06 | 1.249754837 | 0.057826975 |
|  | ZFP90 | 37235 | AA | 2.590496304 | 13.33638886 | 2.239690117 | 79.4124448 | 0.004430622 |
|  | SMC4 | 67484 | AA | -9.662303117 | 6.36E-05 | 1.02E-07 | 0.0396158 | 0.003245325 |
|  | CCDC142 | 54075 | AA | -4.155236985 | 0.015682074 | 0.002230459 | 0.110258672 | 2.97E-05 |
|  | CEP57L1 | 77169 | AA | -3.691654738 | 0.024930714 | 0.003683232 | 0.168748654 | 0.000154545 |
|  | S100A6 | 119889 | AA | -2.076954437 | 0.125311275 | 0.045742479 | 0.343289563 | 5.36E-05 |
|  | TRAF4 | 39978 | AA | -8.58152582 | 0.000187539 | 1.34E-07 | 0.262679316 | 0.020253178 |
|  | ZBTB8A | 1609 | AA | 2.013443768 | 7.48906359 | 1.788099265 | 31.36630865 | 0.005865248 |
|  | RABL2B | 62922 | AA | -2.198511626 | 0.110968198 | 0.008516949 | 1.445815972 | 0.093250838 |
|  | CHD9 | 36413 | AA | 2.031359656 | 7.624445935 | 2.227988482 | 26.09177574 | 0.001211246 |
|  | SRSF7 | 53278 | AA | 13.49508085 | 725837.0704 | 36.2973336 | 14514549707 | 0.007566978 |
| AD | GSTM2 | 4063 | AD | -12.79852315 | 2.76E-06 | 7.51E-09 | 0.001017989 | 2.18E-05 |
|  | RAD51C | 42720 | AD | 6.351749291 | 573.4950418 | 38.32124369 | 8582.61714 | 4.20E-06 |
|  | RPLP2 | 13770 | AD | 2.80715398 | 16.56271326 | 3.071631888 | 89.30870646 | 0.001093322 |
|  | WASH4P | 32775 | AD | 1.408829066 | 4.091162118 | 1.400990249 | 11.94698356 | 0.009976617 |
|  | THAP5 | 81418 | AD | -3.155352582 | 0.04262337 | 0.003253878 | 0.558334316 | 0.016217555 |
|  | GBP3 | 3709 | AD | -0.964200062 | 0.381288085 | 0.160644471 | 0.90498355 | 0.028789965 |
|  | STRA6 | 31688 | AD | -0.689660462 | 0.501746402 | 0.256948263 | 0.97976709 | 0.043401194 |
|  | MOV10L1 | 62785 | AD | -9.486851394 | 7.58E-05 | 1.77E-06 | 0.003245203 | 7.42E-07 |
|  | ECHDC2 | 3029 | AD | 2.025572331 | 7.580448227 | 2.512458079 | 22.87130512 | 0.000324333 |
|  | ZNF23 | 37444 | AD | -1.03167113 | 0.356410854 | 0.14205828 | 0.894201285 | 0.027932446 |
|  | RPS20 | 83891 | AD | -3.333053211 | 0.035683988 | 0.003590361 | 0.354657065 | 0.0044456 |
|  | INPP5B | 1852 | AD | -0.844126738 | 0.429932638 | 0.220359665 | 0.838819905 | 0.013309755 |
| AP | KIAA1598 | 13239 | AP | -11.01928901 | 1.64E-05 | 1.90E-07 | 0.0014101 | 1.25E-06 |
|  | STMN3 | 60140 | AP | -8.630111533 | 0.000178645 | 7.53E-08 | 0.423646337 | 0.029512409 |
|  | GNAQ | 86654 | AP | -6.307468454 | 0.001822642 | 4.09E-06 | 0.811686629 | 0.042660943 |
|  | ZSCAN5A | 52162 | AP | -1.737480838 | 0.175963122 | 0.038822462 | 0.797554272 | 0.024238451 |
|  | FCGR3A | 8673 | AP | 3.273453608 | 26.4023655 | 4.457849452 | 156.3724642 | 0.000309915 |
|  | GTPBP10 | 80387 | AP | -9.85248493 | 5.26E-05 | 7.63E-08 | 0.036273002 | 0.003131009 |
|  | COLEC11 | 52578 | AP | 0.880060527 | 2.411045635 | 1.27698395 | 4.552242849 | 0.006648299 |
|  | CLDN10 | 26114 | AP | -2.736640462 | 0.064787638 | 0.008515577 | 0.49291296 | 0.008211596 |
|  | UBXN2A | 52794 | AP | 2.809219962 | 16.5969669 | 3.908380071 | 70.47915132 | 0.000140396 |
|  | SOGA2 | 44571 | AP | 1.226168433 | 3.408145947 | 1.436460999 | 8.086163709 | 0.005409503 |
|  | MOCS2 | 71995 | AP | -3.592585407 | 0.02752707 | 0.000596916 | 1.269423901 | 0.066074448 |
| AT | FAM124B | 57773 | AT | 2.357082143 | 10.56009362 | 3.100934857 | 35.96192192 | 0.000163184 |
|  | TM4SF19 | 68238 | AT | -2.907413792 | 0.054616798 | 0.004852189 | 0.614772933 | 0.018580855 |
|  | FBF1 | 43530 | AT | -3.782843962 | 0.022757877 | 0.002701285 | 0.191731359 | 0.000503422 |
|  | PTCHD4 | 76445 | AT | 1.542528257 | 4.676398475 | 1.814786381 | 12.05029028 | 0.001403235 |
|  | C16orf46 | 37750 | AT | 1.7208046 | 5.589023586 | 1.583056756 | 19.73219503 | 0.007502231 |
|  | CUX1 | 81071 | AT | 4.396002628 | 81.12592915 | 5.771585183 | 1140.313479 | 0.00111465 |
|  | AP3B2 | 32239 | AT | -0.645854217 | 0.524214558 | 0.2586143 | 1.062589741 | 0.07320337 |
|  | TSTD2 | 87013 | AT | -9.030232634 | 0.000119735 | 1.07E-07 | 0.13418197 | 0.011715026 |
| ES | ALPK1 | 70369 | ES | -23.04613381 | 9.80E-11 | 1.38E-15 | 6.96E-06 | 5.27E-05 |
|  | NOL8 | 86863 | ES | -3.041171081 | 0.047778904 | 0.013455468 | 0.169657693 | 2.55E-06 |
|  | PLEKHG2 | 49826 | ES | -6.999673385 | 0.00091218 | 1.01E-06 | 0.821149593 | 0.043722883 |
|  | GPR124 | 83355 | ES | -7.980866548 | 0.000341943 | 6.78E-07 | 0.172395191 | 0.011948805 |
|  | NT5C2 | 12992 | ES | 2.858953876 | 17.44326957 | 3.429946665 | 88.70915004 | 0.000570453 |
| ME | KHK | 52929 | ME | -12.27089765 | 4.69E-06 | 8.84E-10 | 0.024838261 | 0.005038648 |
|  | H2AFY | 96931 | ME | -1.79875413 | 0.165504957 | 0.03583913 | 0.764301224 | 0.021205642 |
|  | FGFR1 | 133022 | ME | -1.213975775 | 0.297014068 | 0.090087707 | 0.979238562 | 0.046104881 |
|  | EMID1 | 61575 | ME | 0.909400204 | 2.482832894 | 0.695026323 | 8.869389508 | 0.161535998 |
|  | CMC2 | 94205 | ME | -0.584573639 | 0.557343441 | 0.32223707 | 0.96398503 | 0.036512252 |
|  | PTK2 | 98071 | ME | 2.561829516 | 12.95950526 | 1.938075408 | 86.65750355 | 0.008229784 |
|  | P4HA1 | 12122 | ME | -1.461575647 | 0.23187064 | 0.031460236 | 1.708950754 | 0.151531575 |
|  | DNM1 | 87724 | ME | -2.801877002 | 0.060696029 | 0.007369045 | 0.499930196 | 0.009203964 |
| RI | LUC7L3 | 42475 | RI | -10.65324066 | 2.36E-05 | 9.52E-08 | 0.005859809 | 0.000152482 |
|  | AP4B1 | 4309 | RI | 1.315740362 | 3.727509669 | 1.501586903 | 9.253096377 | 0.004564127 |
|  | EXO5 | 2003 | RI | -1.292069321 | 0.274701748 | 0.115725227 | 0.652070878 | 0.00339568 |
|  | ARMC5 | 36260 | RI | -0.878858447 | 0.415256679 | 0.169084263 | 1.019835352 | 0.055222792 |
|  | CACTIN | 46713 | RI | 3.764448282 | 43.13989823 | 4.093007433 | 454.6903103 | 0.001731694 |
|  | CCDC66 | 65347 | RI | -5.769642906 | 0.003120872 | 2.42E-05 | 0.402816364 | 0.01998482 |
|  | XBP1 | 61563 | RI | -2.830522294 | 0.05898204 | 0.010157287 | 0.342500998 | 0.001611418 |
|  | HOXA1 | 79050 | RI | -1.332289038 | 0.263872555 | 0.083328135 | 0.835596829 | 0.023490417 |
|  | CCDC88A | 53613 | RI | 7.479801025 | 1771.88818 | 8.509156373 | 368965.8039 | 0.006032039 |
|  | FOXO4 | 89391 | RI | -17.03360278 | 4.00E-08 | 7.74E-13 | 0.002071315 | 0.002099114 |
| AS, alternative splicing; AA, alternate acceptor site; AD, alternate donor site; AP, alternate promoter; AT, alternate terminator; ES, exon skip; ME, mutually exclusive exons; RI, retained intron | | | | | | | | |
