# Supplementary material for: Identification of Survival-Associated Alternative Splicing Signatures in Lung Squamous Cell Carcinoma
Source: Front Oncol. 2020 Sep 30;10:587343. doi: 10.3389/fonc.2020.587343 (PMC7561379; doi:10.3389/fonc.2020.587343)
Supplement: Supplementary file 3 [file Image_1.PDF]

Supplementary Material

Supplementary Figures

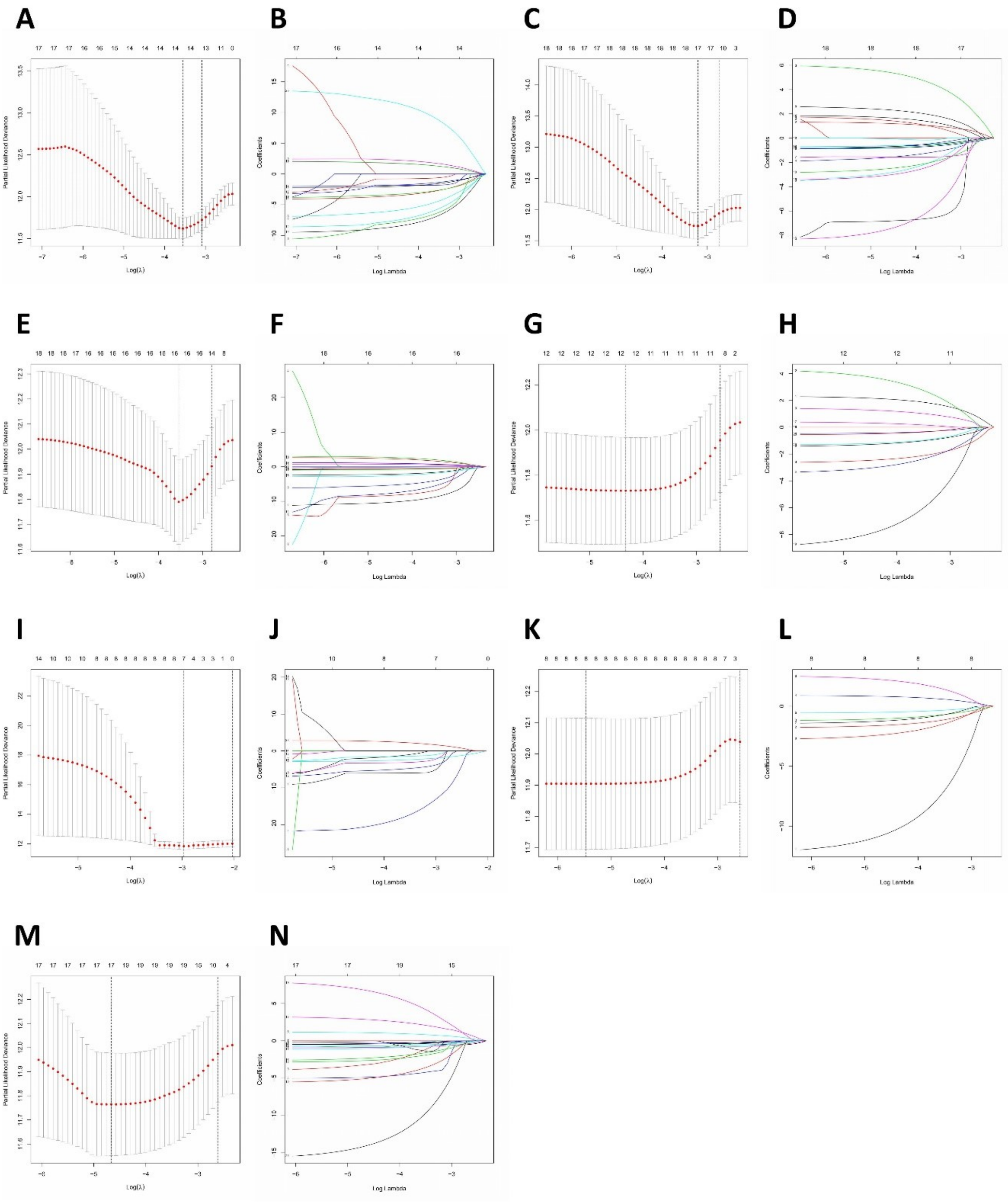

Supplementary Figure 1. LASSO regression analysis of survival-related AS events. AA cohort **(A, B)**, AD cohort **(C, D)**, AP cohort **(E, F)**, AT cohort **(G, H)**, ES cohort **(I, J)**, ME cohort **(K, L)**, and RI cohort **(M, N)**.

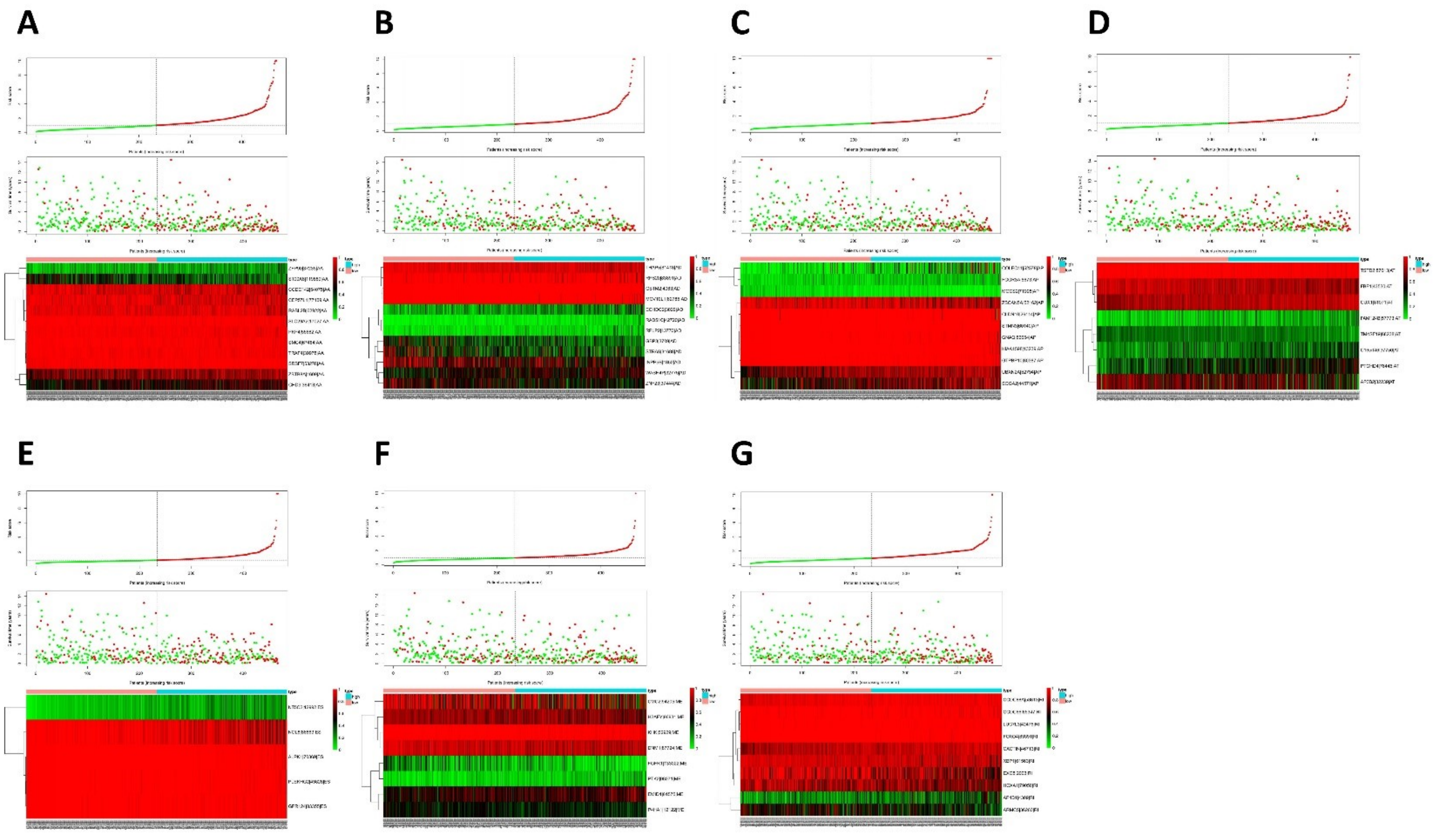

Supplementary Figure 2. Risk scores analyses of prognostic models. The upper part represents the risk score curves, the middle part indicates the distribution of patients' survival time and status, and the bottom shows the PSI value heatmap for the final prognostic model. AA cohort **(A)**, AD cohort **(B)**, AP cohort **(C)**, AT cohort **(D)**, ES cohort **(E)**, ME cohort **(F)**, and RI cohort **(G)**.
